# Association of sodium-glucose cotransporter 2 inhibitors with risk of major adverse cardiovascular events in type 2 diabetes patients with acute coronary syndrome: a propensity score‑matched analysis

**DOI:** 10.1186/s12933-024-02200-7

**Published:** 2024-03-25

**Authors:** Tao Liu, Zeyuan Fan, Bing Xiao, Chang He, Shicong Wang

**Affiliations:** Department of Coronary Heart Disease, Civil Aviation General Hospital, No. 1 Gaojingjia Road, Chaoyang District, Beijing, China

**Keywords:** Sodium-glucose cotransporter 2 inhibitor, Major adverse cardiovascular events, Type 2 diabetes, Acute coronary syndrome, Propensity score‑matched

## Abstract

**Background:**

This study aimed to investigate the association of sodium-glucose cotransporter 2 inhibitors (SGLT2i) use with cardiovascular (CV) clinical outcomes in type 2 diabetes (T2D) patients with acute coronary syndrome (ACS).

**Methods:**

Data of T2D patients hospitalized for ACS at Civil Aviation General Hospital from January 2019 to December 2022 were collected. Based on SGLT2i use or not, patients were stratified as SGLT2i group and SGLT2i-free group. A 1:1 nearest-neighbor propensity score-matched (PSM) was performed to adjust for the confounding factors and facilitate the robust comparisons between groups. The first occurrence of major adverse cardiovascular events (MACE) with 1 year follow-up, which consisted of CV death, all cause death, non-fatal myocardial infarction or stroke, coronary revascularization or heart failure readmission, was assessed. Kaplan–Meier analysis and Cox regressions were conducted to evaluate the prognostic significance of SGLT2i use. Subgroup analyses were performed to assess the interaction between subgroups and SGLT2i use.

**Results:**

A total of 925 patients were included, and the SGLT2i use increased from 9.9% in 2019 to 43.8% in 2022. 226 pairs were finally matched using the PSM model. During 1 year follow-up period, a total of 110 patients experienced MACE in the matched cohort, with a rate of 24.3%. Survival analyses showed cumulative incidence of MACE, CV death, and heart failure readmission in the SGLT2i group were significantly lower than the SGLT2i-free group. Additionally, the adjusted Cox analyses demonstrated that SGLT2i was associated with a 34.1% lower risk of MACE (HR 0.659, 95% CI 0.487–0.892, P = 0.007), which was primarily driven by a decrease in the risk of CV death by 12.0% (HR 0.880, 95% CI 0.7830.990, P = 0.033), and heart failure readmission by 45.5% (HR 0.545, 95% CI 0.332–0.893, P = 0.016). This MACE preventive benefit was consistent across different subgroups (P interaction > 0.05 for all comparisons).

**Conclusions:**

In T2D patients with ACS, there was a clear increasing trend in SGLT2i use. SGLT2i was associated with a significantly lower risk of MACE, driven by the decrease in the risk of CV death, and heart failure readmission. Our study confirmed real-world use and efficacy of SGLT2i in a general T2D population with ACS.

**Supplementary Information:**

The online version contains supplementary material available at 10.1186/s12933-024-02200-7.

## Background

With its gradually rising incidence, diabetes has become one of the major causes of morbidity and mortality worldwide. An estimate shows that the global diabetes prevalence is expected to increase to 10.2% (578 million) by 2030 and 10.9% (700 million) by 2045 [1]. Type 2 diabetes (T2D), the most common diabetes, accounts for approximately 90% of the total patients and various factors can contribute to its rising trend, for example, ageing, obesogenic environment and urbanization. Cardiovascular disease (CVD) has become one of the primary comorbidities of T2D, and accounts for at least 50% of deaths in T2D patients [2, 3]. Acute coronary syndrome (ACS), the most severe type of CVD, includes ST-elevation myocardial infarction (STEMI), non-ST-elevation myocardial infarction (NSTEMI) and unstable angina (UA). People with T2D were reported to have three-fold greater odds to develop ACS [4]. Moreover, ACS mortality for T2D patients were four times higher in male and seven times higher in female population [5]. Meanwhile, tight glycemic control in ACS has been observed to play a cardioprotective effect by various mechanisms including anti-inflammatory and anti-apoptotic activities, anti-oxidative stress, and endothelium protection [6]. Intriguingly, novel antidiabetic agents, for example, sodium-glucose cotransporter 2 inhibitors (SGLT2i), demonstrated a significant improvement in the cardiovascular (CV) outcomes in high-risk subjects without the risk of hypoglycemia, might exert this cardioprotective effect even in acute conditions such as ACS [7]

SGLT2i are oral glucose-lowering agents which can significantly increase the excretion of urinary glucose via inhibiting SGLT2 in the renal proximal tubules [8]. Beyond glycemic control effects, several large cardiovascular outcome trials (CVOTs) have revealed their significant CV benefits, including a decreased risk of CV death and rehospitalization for heart failure [9–11]. Based on these consistent data, both American Diabetes Association (ADA) guidelines and European Association for the Study of Diabetes (EASD) guidelines recommend the use of SGLT2i in T2D patients with established atherosclerotic cardiovascular disease (ASCVD) or with very high or high CV risk [12, 13]. However, all the current CVOTs of SGLT2i excluded patients experiencing ACS within 14 days before enrollment. Therefore, there was still a lack of data on the CV effect of SGLT2i in T2D patients with new onset of ACS. Our current study aimed to utilize real world data to explore whether SGLT2i could improve CV clinical outcomes in T2D patients with ACS.

## Materials

### Study design and participants

This study was a single-center retrospective study. Data of T2D patients who underwent coronary angiography for ACS at the Cardiology Department of the Civil Aviation General Hospital, were collected from January 2019 to December 2022. Inclusion criteria were as follows: (1) age ≥ 18 years; (2) hospitalized for ACS, including STEMI, NSTEMI, or UA. STEMI diagnosis was defined per 2019 Chinese Society of Cardiology guidelines for the diagnosis and management of patients with STEMI [14], while diagnosis of NSTEMI and UA were based on 2016 Guidelines and consensus for the management of patients with non-ST-elevation ACS [15]; and (3) T2D patients diagnosed based on ADA guidelines (2020 edition) [16]. Exclusion criteria were (1) previous use of SGLT2i; (2) cardiogenic shock; (3) severe heart failure (Killip class ≥ III); (4) malignant ventricular arrhythmias; (5) severe hepatic dysfunction (serum alanine transaminase levels exceeding 3 times the upper normal limit) or renal dysfunction (serum creatinine surpassing 221 μmol/l or estimated glomerular filtration rate (eGFR) less than 30 ml/min/1.73 m^2^); (6) perioperative cardiopulmonary resuscitation; and (7) malignant tumors. The study got approval by the Ethics Committee of the Civil Aviation General Hospital (2022⁃L-K⁃33) and was conducted in accordance with Declaration of Helsinki guidelines. Patient informed consent was waived as part of the study approval.

Based on the electronic medical records, we collected clinical, laboratory, and clinical outcome data for all participants. Patients’ clinical baseline demographic data included age, gender, height, weight, blood pressure, heart rate, smoking, diabetes duration, hyperlipidemia, hypertension, chronic heart failure, valvular heart disease, and in-hospital medication. Prior to study procedures, laboratory indices, including cardiac troponin I (cTnI), glycated hemoglobin (HbA1c), high-sensitivity C-reactive protein (hs-CRP), low-density lipoprotein (LDL), high-density lipoprotein (HDL), triglycerides (TG), total cholesterol (TC), B-type natriuretic peptide (BNP), and eGFR, were recorded. Antidiabetic agents, including SGLT2i, glucagon-like peptide-1 receptor agonists (GLP-1RAs), metformin, DPP-4 inhibitors and insulin, were documented.

### SGLT2i exposure

According to the exposure of SGLT2i, patients were divided into the SGLT2i-free group and the SGLT2i group. The SGLT2i group consisted of patients receiving initial administration of SGLT2i during ACS hospitalization and the follow-up period. SGLT2i-free users were defined as the subjects who were SGLT2i nonusers. Medication history of patients included was obtained from prescriptions in the electronic medical record.

### CV clinical outcomes

Time to first major adverse cardiovascular events (MACE) within 1 year after discharge and its individual components were assessed in the study. MACE was defined as the composite of CV death, all cause death, non-fatal myocardial infarction or stroke, coronary revascularization or heart failure readmission. To ascertain the occurrence of MACE, electronic medical records, including clinic visits and telephone interviews, were comprehensively reviewed.

### Statistical analyses

Continuous data were described as means ± standard deviations or medians (interquartile range) based on the normality of the distribution, which was determined based on the Kolmogorov–Smirnov test. Categorical variables were presented using frequencies and percentages, and differences between groups were analyzed using chi-square tests or Fisher’s exact tests.

Propensity score-matched (PSM) analysis was developed to reduce confounding biases in observational studies. A 1-to-1 match was established using PSM analysis with a caliper width of 0.20. Propensity scores were generated to estimate the likelihood of SGLT2i use, based on multivariable logistic regression models. Age, gender, BMI, smoking, T2D duration, comorbidities including hypertension, hyperlipidemia, and chronic heart failure, the use of antidiabetic medications such as insulin and GLP-1RAs, ACS type, PCI and extent of coronary artery disease, were included as the factors for matching. Absolute standardized differences were computed for all baseline characteristics. A standardized difference of 10% was considered to achieve balance and indicate comparability between groups.

Cumulative event rates over time were obtained by the Kaplan–Meier method and the differences between groups were assessed by log-rank test. Cox proportional hazard analysis was conducted to estimate the hazard ratio with 95% confidence intervals for various endpoint events. Notably, subgroup analyses were performed by including subgroup as a fixed factor to evaluate the interaction between subgroup and SGLT2i use. Statistical significance was considered as a two-tailed p-value < 0.05. The statistical analyses were carried out using R software (version 4.3.2).

## Results

### Baseline characteristics of the full cohort

**Fig. 1:**
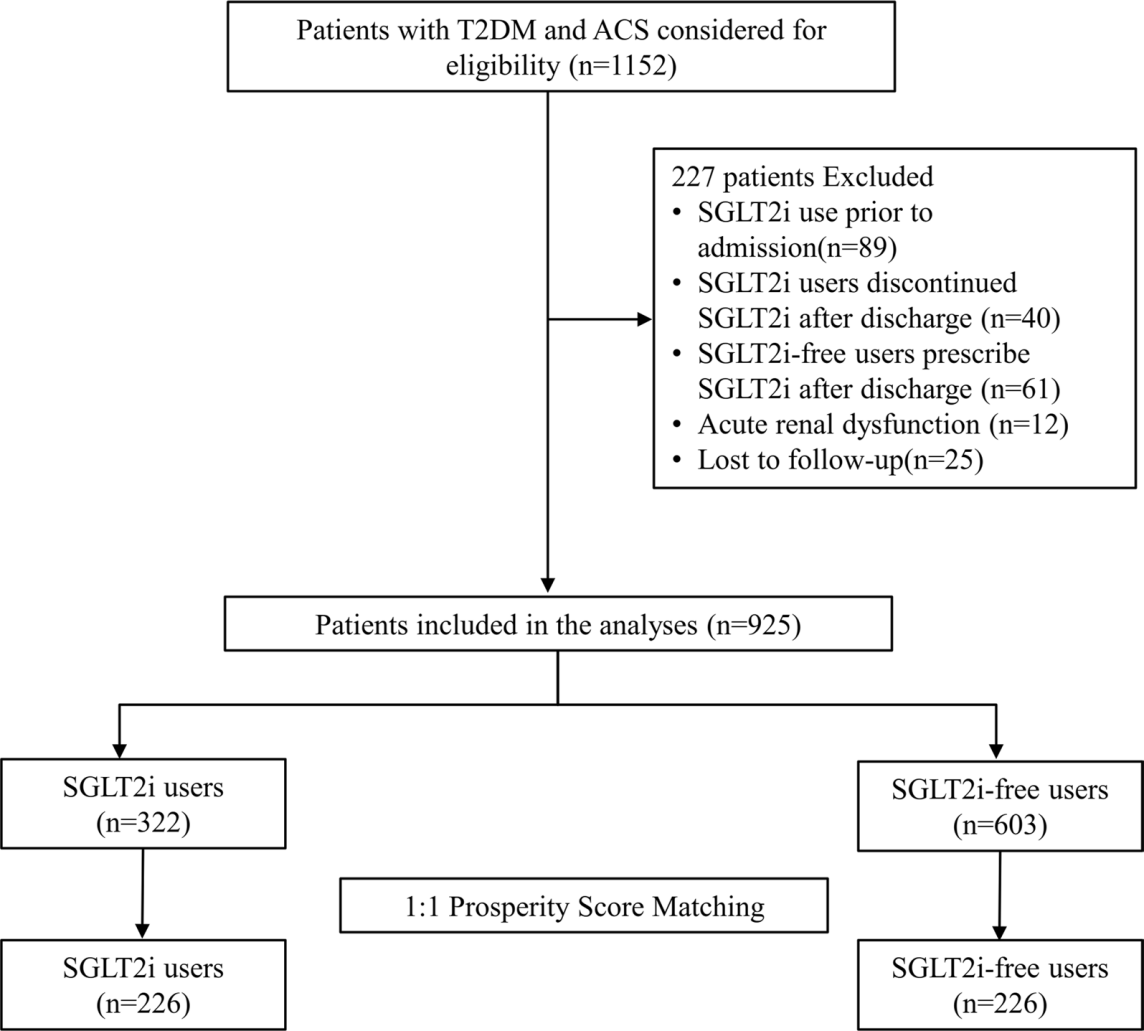
Study flowchart to illustrate the study selection and follow-up. T2D, type 2 diabetes; ACS, acute coronary syndrome

**Fig. 2:**
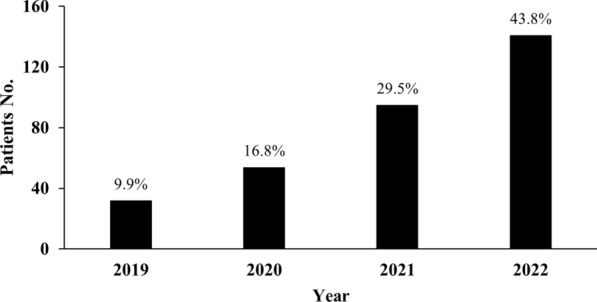
Patients enrollment per year

From January 2019 to December 2022, 1152 T2D patients admitted to Civil Aviation General Hospital for ACS were retrospectively considered for participation in our study. After 1 year follow-up, a total of 925 patients were finally enrolled in our study, whereas 202 patients were excluded based on the criteria and 25 patients were lost to follow up (Fig. 1). Among the SGLT2i patients, 9.9% out of 322 patients were enrolled in the year 2019, 16.8% in the year 2020, 29.5% in the year 2021 and 43.8% in the year 2022, which indicated an increasing SGLT2i use trend in the full cohort (Fig. 2).

Of the 925 patients, 650 (70.3%) patients had a history of hypertension, 452 (48.9%) had hyperlipidemia, and 393 (42.5%) had chronic heart failure and 21 (2.3%) patients were combined with valvular heart disease. The average age was 62.3 years with an average body mass index (BMI) of 28.3 kg/m^2^, 61.4% were males, and the average HbA1c was 7.48%. Baseline characteristics of the two groups were summarized in Table 1. Compared with those in the SGLT2i-free group, patients in the SGLT2i group demonstrated to be with older ages, bigger BMI, higher levels of hs-CRP and BNP, and the proportion of men was higher. Regarding the use of other medications including antidiabetic agents, there was no significant difference, except β-blockers and GLP-1RAs, between the two groups.

**Table 1:** Clinical characteristics of the full cohort and PSM cohort

Variables	Full cohort			PSM cohort		
	SGLT2i Group(n = 322)	SGLT2i-free Group(n = 603)	*P* value	SGLT2i Group(n = 226)	SGLT2i-free Group(n = 226)	*P* value
Age (years)	63.5 ± 9.8	61.7 ± 12.6	0.03	62.9 ± 10.8	62.1 ± 11.7	0.45
Male, n (%)	212 (65.8)	350 (58)	0.02	143 (63.3)	129 (57.1)	0.18
Current smoking, n (%)	109 (33.9)	210 (34.8)	0.77	86 (38.1)	76 (33.6)	0.33
BMI (kg/m^2^)	28.9 ± 7.8	27.3 ± 6.3	<0.001	28.9 ± 7.5	27.8 ± 7.3	0.11
Diabetes Duration (years)	8.65 ± 2.56	8.31 ± 2.78	0.07	8.59 ± 2.81	8.21 ± 2.75	0.15
Other comorbidities, n (%)						
Hypertension	218 (67.7)	432 (71.6)	0.21	165 (73)	150 (66.4)	0.12
Hyperlipidemia	162 (50.3)	290 (48.1)	0.52	114 (50.4)	110 (48.7)	0.71
Chronic heart failure	127 (39.4)	266 (44.1)	0.17	86 (38.1)	97 (42.9)	0.29
Valvular heart disease	8 (2.5)	13 (2.2)	0.75	6 (2.7)	5 (2.2)	0.76
Laboratory variables						
Hemoglobin (g/L)	131.7 ± 39.6	135.6 ± 38.9	0.15	132.1 ± 40.7	134.9 ± 39.1	0.46
HbA1c (%)	7.65 ± 2.85	7.39 ± 3.73	0.28	7.5 ± 3.12	7.3 ± 3.81	0.41
hs-CRP (mg/L)	2.67 ± 0.63	2.57 ± 0.56	0.01	2.71 ± 0.65	2.61 ± 0.66	0.11
FPG (mmol/L)	8.8 ± 2.25	8.6 ± 2.73	0.29	8.82 ± 2.12	8.59 ± 2.63	0.31
TC (mmol/L)	4.22 ± 1.15	4.09 ± 1.21	0.11	4.32 ± 1.31	4.15 ± 1.22	0.15
LDL (mmol/L)	2.59 ± 1.32	2.73 ± 1.21	0.10	2.65 ± 1.32	2.73 ± 1.11	0.49
Peak cTnI (ug/L)	39.6 (16.7, 59.8)	33.6 (15.8, 56.9)	0.13	36.9 (18.5, 53.9)	35.9 (20.9, 51.8)	0.16
BNP (ug/L)	89.8 ± 37.9	83.9 ± 40.8	0.03	88.7 ± 38.1	85.6 ± 39.9	0.40
SCr (umol/L)	105.5 ± 26.9	108.6 ± 22.7	0.06	106.7 ± 25.8	108.5 ± 23.5	0.44
eGFR (ml/min/1.73 m^2^)	69.8 ± 22.8	67.3 ± 23.4	0.12	69.5 ± 22.7	67.1 ± 23.3	0.27
Left ventricular ejection fraction (%)	51.2 ± 13.3	52.7 ± 13.9	0.11	51.6 ± 12.6	52.1 ± 13.8	0.69
ACS Type, n (%)			0.16			0.72
STEMI	139 (43.2)	291 (48.3)		104 (46.0)	110 (48.7)	
NSTEMI	108 (33.5)	201 (33.3)		68 (30.1)	69 (30.5)	
UA	75 (23.3)	111 (18.4)		54 (23.9)	47 (20.8)	
Medications						
Metformin	215 (66.8)	432 (71.6)	0.12	163 (72.1)	175 (77.4)	0.19
Insulin	172 (53.4)	359 (59.5)	0.07	119 (52.7)	123 (54.4)	0.71
ACEI/ARB	182 (56.5)	376 (62.4)	0.08	121 (53.5)	129 (57.1)	0.45
Calcium channel blockers	106 (32.9)	229 (38)	0.13	75 (33.2)	80 (35.4)	0.62
β-Blockers	182 (56.5)	390 (64.7)	0.02	123 (54.4)	132 (58.4)	0.39
Statins	315 (97.8)	588 (97.5)	0.77	215 (95.1)	219 (96.9)	0.34
GLP-1RAs	40 (12.4)	118 (19.6)	0.01	25 (11.1)	39 (17.3)	0.06
Aspirin	322 (100)	603 (100)	/	226 (100)	226 (100)	/
P2Y12 inhibitors			0.12			0.45
Ticagrelor	178 (55.3)	301 (49.9)		115 (50.9)	123 (54.4)	
Clopidogrel	144 (44.7)	302 (50.1)		111 (49.1)	103 (45.6)	
Procedure characteristics						
PCI, n (%)	258 (80.1)	492 (81.6)	0.59	186 (82.3)	181 (80.1)	0.55
Extent of CAD, n (%)			0.13			0.12
Any left main disease	9 (2.8)	12 (2.0)		6 (2.7)	8 (3.5)	
1-vessel disease	48 (14.9)	98 (16.3)		18 (8.0)	29 (12.8)	
2-vessel disease	130 (40.4)	201 (33.3)		81 (35.8)	91 (40.3)	
≥3-vessel disease	135 (41.9)	292 (48.4)		121 (53.5)	98 (43.4)	

ACEI/ARB, angiotensin converting enzyme inhibitor/angiotensin receptor blocker; BMI, body mass index; BNP, B-type natriuretic peptide; CAD, coronary artery disease; CAG, coronary angiography; cTnI, cardiac troponin I; eGFR, estimated glomerular filtration rate; FPG, fasting plasma glucose; hs-CRP, high-sensitivity C-reactive protein; LDL, low-density lipoprotein cholesterol; PCI, percutaneous coronary intervention; TC, total cholesterol, UA, unstable angina

### Baseline characteristics of the PSM cohort

To minimize confounding bias with respect to baseline characteristics, we identified 226 SGLT2i users who were ultimately matched with 226 SGLT2i-free users using 1-to-1 matching. Of the 452 patients, 315 (69.7%) had a history of hypertension, 224 patients (49.6%) had hyperlipidemia, and 183 (40.5%) had chronic heart failure. The mean age was 62.5 years with an average BMI of 28.4 kg/m^2^, 60.2% were males, and the average HbA1c was 7.40%. Comparison results summarized in Table 1 showed a similar distribution of baseline characteristics between the two groups. In addition, the absolute standardized differences were < 10%, which exhibited the balance between the two groups was appropriate.

### CV clinical outcomes

**Fig. 3:**
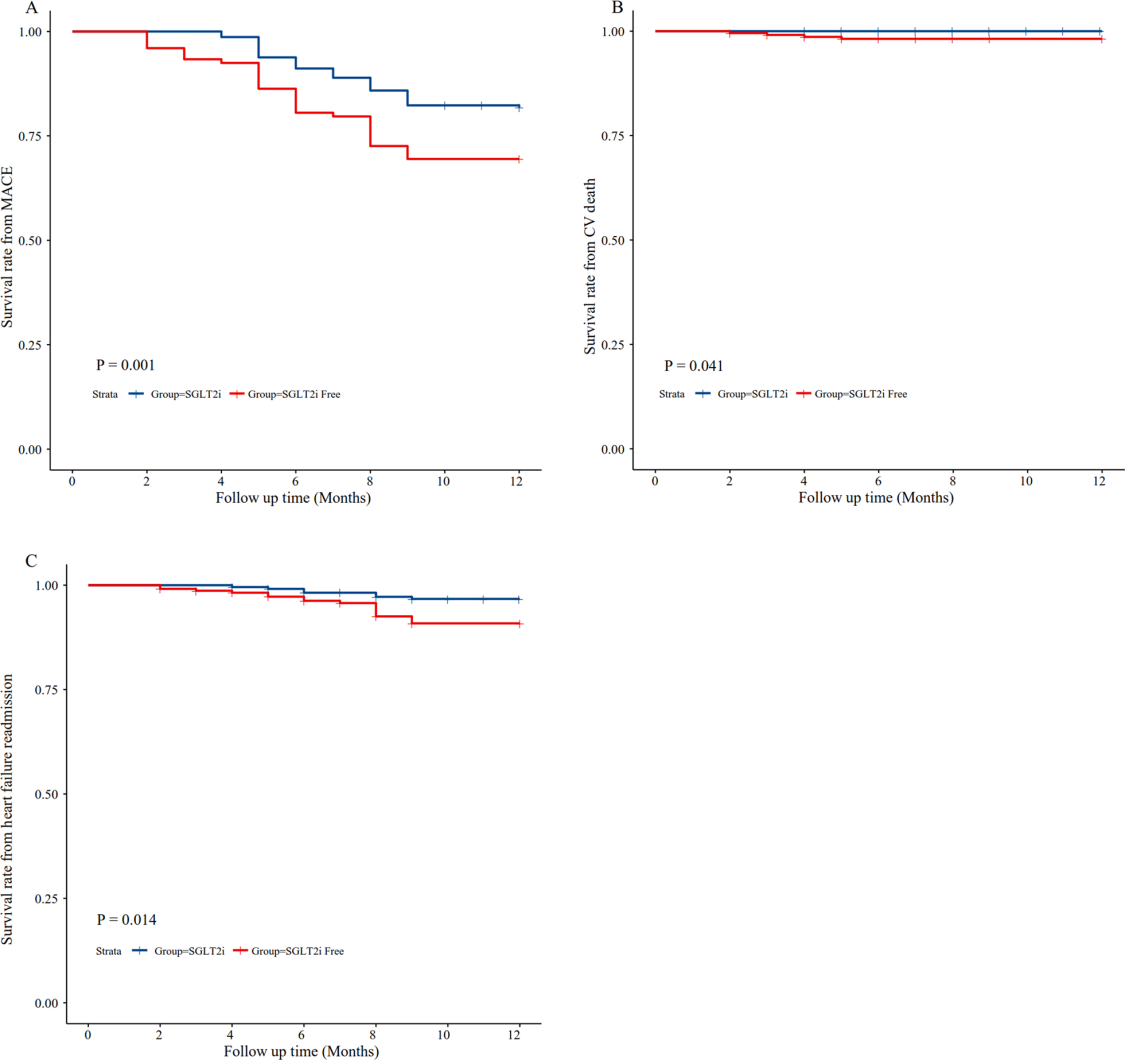
Kaplan–Meier curves to plot the cardiovascular outcomes in the matched population. **A**. Kaplan–Meier curve for MACE. **B**. Kaplan–Meier curve for CV death. **C** Kaplan–Meier curves for heart failure readmission. MACE, major adverse cardiac event; CV, cardiovascular

During 1 year follow-up period, a total of 110 patients experienced MACE in the matched cohort, with a rate of 24.3%. MACE were reported in 41 out of 226 patients in the SGLT2i group and 69 patients in the SGLT2i-free group (HR 0.545, 95% CI 0.370–0.803, P = 0.001) (Fig. 3A). CV death was documented in 1 patient in the SGLT2i group and 4 patients in the SGLT2i-free group (HR 0.798, 95% CI 0.640–0.995, P = 0.041) (Fig. 3B). Heart failure readmissions were found in 7 patients in the SGLT2i group and 18 patients in the SGLT2i-free group (HR 0.462, 95% CI 0.236–0.902, P = 0.014) (Fig. 3C). Although no statistical difference was demonstrated in terms of all-cause death (Additional file 1: Fig. S1A), recurrent MI (Additional file 1: Fig S1B), non-fatal stroke (Additional file 1: Fig. S1C) or revascularization (Additional file 1: Fig S1D), SGLT2i-free group suffered more events. These results persisted in fully adjusted Cox regression models, which showed that SGLT2i reduced the risk of MACE in T2DM patients with ACS by 34.1% (HR 0.659, 95% CI 0.487–0.892, P = 0.007), primarily driven by a decrease in the risk of CV death by 12.0% (HR 0.880, 95% CI 0.783–0.990, P = 0.033) and heart failure readmission by 45.5% (HR 0.545, 95% CI 0.332–0.893, P = 0.01) (Table 2).

**Table 2:** CV clinical outcomes in the PSM cohort

Outcomes	SGLT2i	SGLT2i-free	Unadjusted Model			Model 1			Model 2		
			HR	95% CI	P Value	Adjusted HR	95% CI	P Value	Adjusted HR	95% CI	P Value
MACE	41(18.1)	69(30.5)	0.545	0.370–0.803	0.002	0.657	0.483–0.893	0.007	0.659	0.487–0.892	0.007
All-cause Death	2(0.9)	5(2.2)	0.783	0.514–1.191	0.253	0.763	0.561–1.040	0.087	0.763	0.560–1.039	0.086
CV death	1(0.4)	4(1.8)	0.798	0.640–0.995	0.045	0.878	0.779–0.990	0.033	0.880	0.783–0.990	0.033
Heart failure readmission	7(3.1)	18(8.0)	0.462	0.236–0.902	0.024	0.543	0.330–0.894	0.016	0.545	0.332–0.893	0.016
Recurrent MI	10(4.4)	14(6.2)	0.801	0.477–1.345	0.401	0.638	0.099–4.093	0.635	0.637	0.099–4.096	0.635
Stroke	12(5.3)	17(7.5)	0.833	0.573–1.210	0.337	0.748	0.381–1.471	0.400	0.748	0.380–1.471	0.400
Revascularization	10(4.4)	15(6.6)	0.751	0.434–1.297	0.304	0.914	0.417–2.004	0.822	0.913	0.414–2.014	0.822

CI, confidence level; CV, cardiovascular; MACE, major adverse cardiovascular events; MI, myocardial infarction; HR, hazard ratio; SGLT2i, Sodium-glucose cotransporter 2 inhibitor

Model 1: Adjusted for age, gender, diabetes duration, hypertension history, hyperlipidemia, chronic heart failure, percutaneous coronary intervention (PCI), acute coronary syndrome (ACS) type, and extent of ACS disease

Model 2: Adjusted for covariates in model 1 and GLP-1RA and eGFR

### Subgroup analyses

**Fig. 4:**
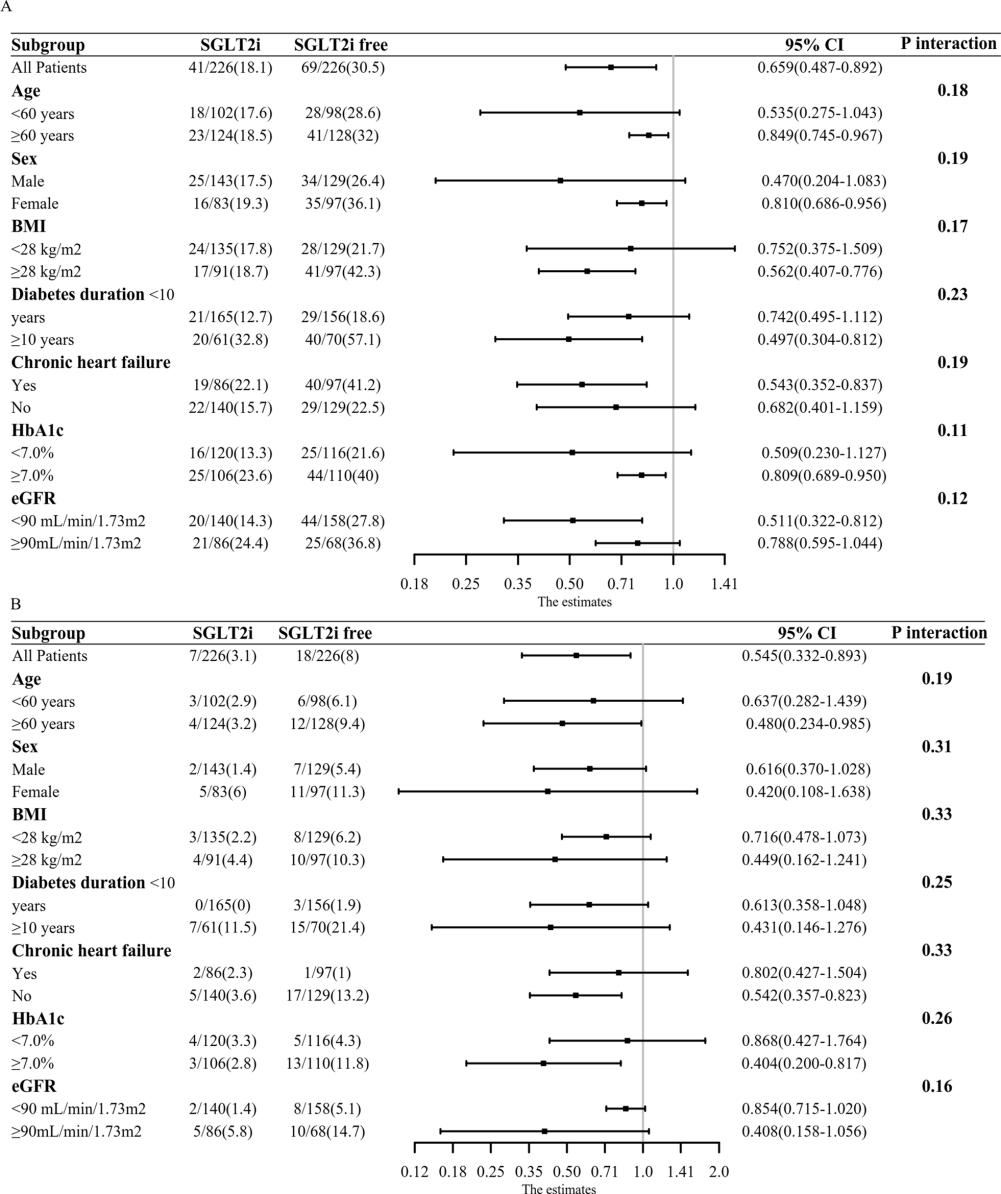
Subgroup forest plots of T2D patients with ACS prescribing SGLT2i vs SGLT2i-free. **A** Subgroup forest plot for major adverse cardiac events (MACE). **B** Subgroup forest plot for heart failure readmission. ACS, acute coronary syndrome; BMI, body mass index; eGFR, estimated glomerular filtration rate; SGLT2i, sodium-glucose cotransporter 2; T2D, type 2 diabetes

We used subgroup analyses to explore whether SGLT2i exerted consistent CV benefits across different patient categories. In subgroup analyses on the SGLT2i lowering risk of MACE, no significant interactions were observed across different categories in terms of age, gender, BMI, diabetes duration, chronic heart failure, eGFR, or HbA1c (P interaction > 0.05 for all comparisons) (Fig. 4A). Thus, SGLT2i benefits in reducing MACE risks were consistently observed across subgroups.

Moreover, we considered evaluating subgroup analyses of CV death and heart failure readmission. Figure 4B showed SGLT2i consistently reduced the risk of heart failure readmission across different categories (P interaction > 0.05 for all comparisons). Due to the extremely limited number of CV death, we didn’t perform subgroup analyses for CV death.

## Discussion

The present study investigated the SGLT2i use trend as well as its impact on MACE in Chinese T2D patients with ACS. Our data showed a clear increasing trend in SGLT2i use among Chinese patients with T2D and ACS from the year 2019 to 2022. Thereafter, to ensure the robustness of our results, we used a PSM model to investigate the association of SGLT2i use with CV clinical outcomes. Our data revealed that SGLT2i was associated with a significant 34.1% lower risk of MACE in T2D patients with ACS. This CV benefit of SGLT2i use was principally driven by a significant decrease in the risk of CV death by 12.0% and heart failure readmission by 45.5%. In addition, SGLT2i effects for lowering CV risk were consistently observed across subgroups.

Our study revealed a significant increasing trend of SGLT2i use in T2D combined with ACS from 9.9% in 2019 to 43.8% in 2022. This finding was consistent with the overall dramatic rise in SGLT2i use in China. A real-world study which enrolled a total of 181,743 prescriptions of SGLT2i in China and showed the annual number of prescriptions of SGLT2i dramatically increased to approximately 140 folds from 2018 to 2021 [17]. The massive increase was closely associated with robust evidence showing the beneficial effects of SGLT2i in T2D with CVD. In addition, the recent increasing trend might be relevant with heart failure indication of SGLT2i approved in China in 2022.

A substantial amount of epidemiological data has firmly established that T2D is relevant with a greater burden of atherosclerosis [18]. In contrast, there is still lack of consensus on the positive effect of intensive glucose management in comparison to non-intensive glucose control on CV outcomes. The Action to Control Cardiovascular Risk in Diabetes (ACCORD) study data showed that the intensive lowering blood glucose was associated with an increased risk of death [19]. As one of most widely used anti-diabetic medications decades ago, rosiglitazone was relevant with a significant increase in the risk of myocardial infarction and with an increase in the risk of CV death [20]. Therefore, CVOTs on novel T2D drugs including SGLT2i, have been requested to detect their CV risk. Several large CVOTs have supported SGLT2i benefits in improving clinical CV outcomes. A recent meta-analysis of 11 randomized controlled trials with a total of 34,058 cases analyzed [21] also verified SGLT2i administration statistically decreased the MACE rate and indicated the effective role of SGLT2i in primary and secondary prevention of CV outcomes, in population regardless of prior coronary heart disease. But, to date, all these studies excluded patients experiencing acute ACS events prior to enrollment. However, T2D patients combined with ACS are at extremely high risk of worse prognosis [22]. According to China CCC-ACS project, 37.6% of Chinese ACS patients had diabetes/possible diabetes and these patients were with a two-fold higher risk of all-cause death and a 1.5-fold higher risk of MACE [23]. Thus, it’s essential to investigate the treatment strategies for such patients. Our current study utilized real world evidence to generate data of SGLT2i use in T2D patients with ACS.

Our study was the first study to report that SGLT2i could reduce the MACE risk by 34.1% in T2D patients with ACS, including a 12.0% lower risk of CV death and 45.5% lower risk of heart failure readmission. This finding agreed with previous SGLT2i data in other populations. Like EMPA-REG OUTCOME trial, the 1st randomized, double-blind, placebo-controlled trial, showed SGLT2i could decrease risks of CV death by 38%, and hospitalization for heart failure by 35% in 7,020 T2D patients at high risk for CV events [24]. Furthermore, CANVAS found that SGLT2i could lower risk of three-point MACE by 14% in 10,142 T2D patients who were at an elevated risk of CVD [25]. Recently, Mao L et al. [26] reported that dapagliflozin could reduce risk of heart failure rehospitalization in diabetic acute myocardial infarction patients.

Several potential actions can be suggested to elucidate the beneficial effects of SGLT2i on MACE, although the precise mechanisms for CV protective effects of SGLT2i remains to be clarified. SGLT2i have exhibited systemic benefits, especially including hemodynamic effects, and metabolic effects, which are mainly led by the promotion of natriuresis and glycosuria and thereafter contribute to their cardioprotective actions. SGLT2i hemodynamic effects are possibly achieved with their antihypertensive activities by reductions of both systolic and diastolic blood pressure with 3–5 mmHg and 2–3 mmHg, respectively [27], without a compensatory heart rate increase. Several mechanisms have been proposed to underline the antihypertensive effects of SGLT2i, including a decrease in sodium reabsorption in proximal renal tubule, accompanied with body weight reduction and improved vascular function reflected in the reductions in arterial stiffness and vascular resistance [28]. The blood pressure modulation by SGLT2i would reduce cardiac preload and afterload, which could prevent patients from CV events, especially CV death. Metabolic effects of SGLT2i have also been reported, independent of their glucuretic actions, which could improve insulin resistance, suppress insulin secretion by beta cells, and stimulate glucagon secretion by alpha cells of pancreatic islets [29]. Additionally, all three SGLT2i have exerted body weight loss effect in T2D patients, mainly by reducing visceral adipose tissue, further contributing to ameliorating cardiometabolic risk profile [30]. Besides systemic effects of SGLT2i, several molecular mechanisms underlining direct cardiac effects of SGLT2i have been reported. Recent experimental studies showed SGLT2i might downregulate Na^+^/H^+^ exchanger and improve Ca^2+^ handling to prevent cardiac remodeling, necrosis, and hypertrophy [31]. Also, the significant reduction of intracellular sodium levels induced by SGLT2i could improve cardiac contractility and dysfunction, which has been proposed as the crucial molecular mechanism of action of SGLT2i to exert a cardioprotective role, particularly in reducing heart failure risk [32]. SGLT2i can also activate AMP-activated protein kinase (AMPK), which has been proved to be disturbed in diabetes patients. Animal studies showed restoration of AMPK signaling would protect mitochondrial function, followed by improved vascular barrier function, and ameliorate cardiac fibrosis and inflammation [33]. Another potential target of SGLT2i is TGF-β/Smad, which is highly engaged in cardiac fibrosis. SGLT2i have reported to inhibit TGF-β/Smad signaling pathway to suppress fibroblast activation and protect myocardial tissues from fibrotic transformation [34]. Multiple encouraging preclinical findings and clinical investigations have revealed that SGLT2i has beneficial effects on coronary microvascular dysfunction which leads to impaired regulation of blood flow and impacts patients’ quality and duration of life [35]. Furthermore, SGLT2i exhibit a positive influence on oxidative stress markers, as well as anti- and pro-apoptotic factors to facilitate the reduction of CV events risk [36].

Subgroup analyses suggested SGLT2i lowering risk of MACE was consistent across different categories, including age, sex, BMI, diabetes duration, chronic heart failure, and levels of HbA1c and eGFR. Additionally, SGLT2i exerted a consistently protective effect on heart failure readmission in T2DM patients with ACS. Our study was consistent with previous reports supporting SGLT2i CV benefits in the general patient population [37-39].

We found no significant difference in the risk of nonfatal myocardial infarction, stroke, and ischemia-driven revascularization between the two groups. The reason may be attributed to SGLT2i mode of action primarily inducing fluid loss from natriuresis and glycosuria, but largely had no impact on the anti–atherogenic effects. Such finding was consistent with recent meta-analysis which also suggested SGLT2i could reduce MACE without any discernable significant reduction of the incidence of MI or stroke [40].

Overall, it’s the first time to show SGLT2i could lower risk of MACE, CV death and heart failure readmission in the T2D patients with ACS. However, there are several limitations in our study. Firstly, the current study was a single- center retrospective study with limited sample size. But to minimize the study bias, PSM analysis was adopted for correction of potential confounders. Furthermore, SGLT-2i free users in our study also administrated different kinds of antidiabetic agents, the effects of which were not studied separately. In the future, prospective large-scale clinical studies with longer follow-up periods are expected to confirm the positive effects of SGLT2i in T2D patients with ACS.

## Conclusion

In T2D patients with ACS, there was a clear increasing trend in SGLT2i use in our hospital from the year 2019 to 2022. SGLT2i was associated with a significant lower risk of CV outcomes driven by a significant decrease in the risk of CV death, and heart failure readmission. Our study confirmed the real-world use and efficacy of SGLT2i in a general T2D population with ACS.

### Supplementary Information

**Additional file 1: Figure S1.** Kaplan–Meier curves to plot the cardiovascular outcomes in the matched population. **A** Kaplan–Meier curve for all-cause death. **B** Kaplan–Meier curve for MI. **C** Kaplan–Meier curves for stroke. **D** Kaplan–Meier curve for revascularization. **E** Kaplan–Meier curve for stroke. MI, myocardial infarction.

## Data Availability

Due to privacy and ethical constraints, the datasets generated and analyzed in this study are not publicly available but can be reasonably requested from the corresponding author.

## Abbreviations

ACCORD: Action to Control Cardiovascular Risk in Diabetes
ACEI/ARB: Angiotensin converting enzyme inhibitor/angiotensin receptor blocker
ACS: Acute coronary syndrome
ADA: American Diabetes Association
AMPK: AMP-activated protein kinase
ASCVD: Atherosclerotic cardiovascular disease
BMI: Body mass index
BNP: B-type natriuretic peptide
CAD: Coronary artery disease
CAG: Coronary angiography
cTnI: Cardiac troponin I
CV: Cardiovascular
CVD: Cardiovascular disease
CVOTs: Cardiovascular outcome trials
EASD: European Association for the Study of Diabetes
eGFR: Estimated glomerular filtration rate
FPG: Fasting plasma glucose
GLP-1RAs: Glucagon-like peptide-1 receptor agonists
HbA1c: Glycated hemoglobin
HDL: High-density lipoprotein
HR: Hazard ratio
hs-CRP: High-sensitivity C-reactive protein
LDL: Low-density lipoprotein cholesterol
MACE: Major adverse cardiovascular event
MI: Myocardial infarction
NSTEMI: Non-ST-elevation myocardial infarction
PCI: Percutaneous coronary intervention
PSM: Propensity score-matched
SGLT2i: Sodium-glucose cotransporter 2 inhibitor
STEMI: ST-elevation myocardial infarction
T2D: Type 2 diabetes
TC: Total cholesterol
TG: Triglycerides
UA: Unstable angina
