# Supplementary figures and images for: Association of sodium-glucose cotransporter 2 inhibitors with risk of major adverse cardiovascular events in type 2 diabetes patients with acute coronary syndrome: a propensity score‑matched analysis
Source: Cardiovasc Diabetol. 2024 Mar 25;23:106. doi: 10.1186/s12933-024-02200-7 (PMC10964669; doi:10.1186/s12933-024-02200-7)

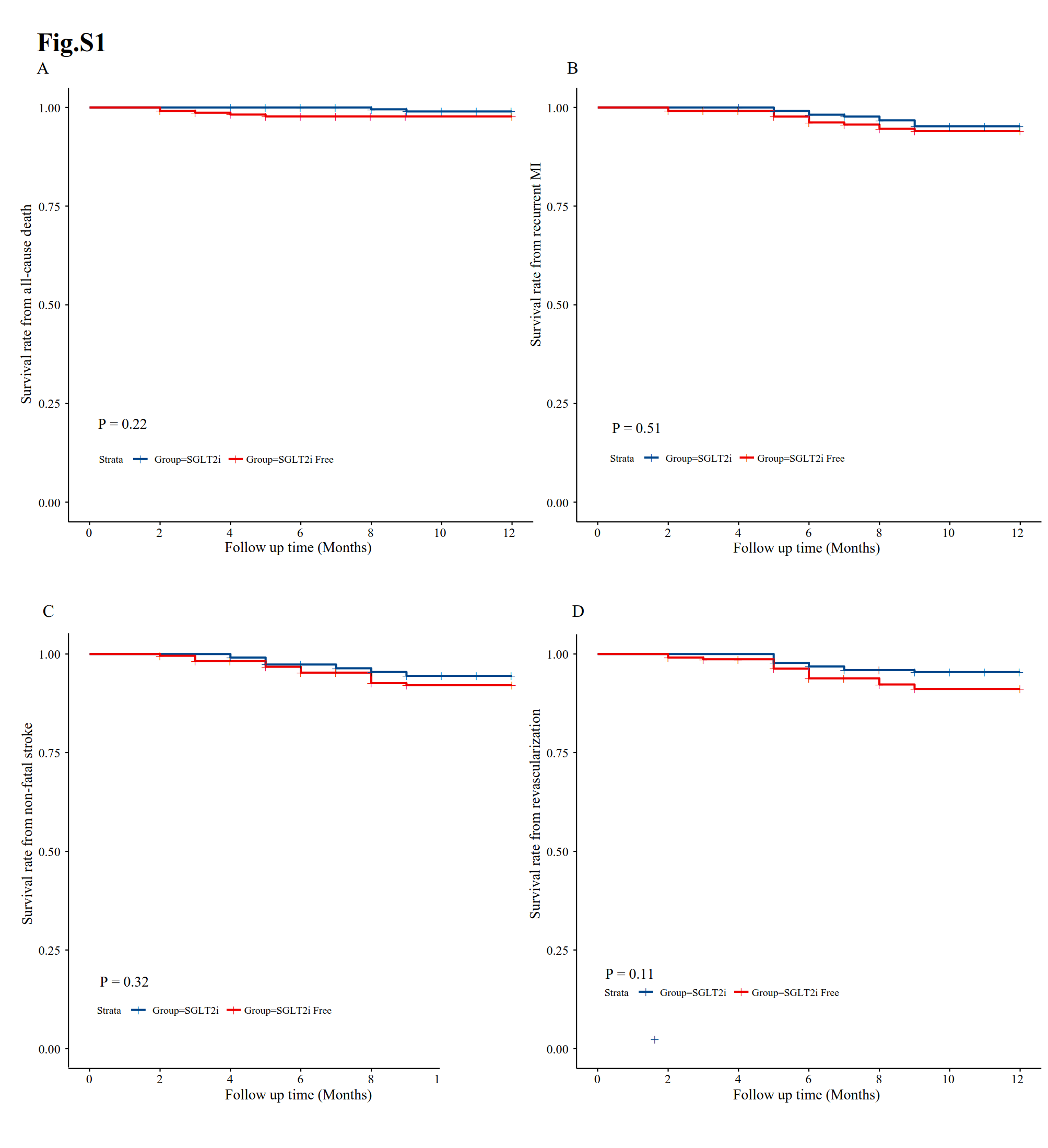

Supplement: Supplementary file 1 — Additional file 1: Figure S1. Kaplan–Meier curves to plot the cardiovascular outcomes in the matched population. A Kaplan–Meier curve for all-cause death. B Kaplan–Meier curve for MI. C Kaplan–Meier curves for stroke. D Kaplan–Meier curve for revascularization. E Kaplan–Meier curve for stroke. MI, myocardial infarction. [file 12933_2024_2200_MOESM1_ESM.tiff]
